# Development of Digital Image Processing as an Innovative Method for Activated Sludge Biomass Quantification

**DOI:** 10.3389/fmicb.2020.574966

**Published:** 2020-09-18

**Authors:** Hashem Asgharnejad, Mohammad-Hossein Sarrafzadeh

**Affiliations:** School of Chemical Engineering, College of Engineering, University of Tehran, Tehran, Iran

**Keywords:** activated sludge, biomass quantification, cell concentration, image processing, RGB analysis

## Abstract

Activated sludge process is the most common method for biological treatment of industrial and municipal wastewater. One of the most important parameters in performance of activated sludge systems is quantitative monitoring of biomass to keep the cell concentration in an optimum range. In this study, a novel method for activated sludge quantification based on image processing and RGB analysis is proposed. According to the results, the intensity of blue color in the macroscopic image of activated sludge culture can be a very accurate index for cell concentration measurement and R^2^ coefficient, Root Mean Square Error (RMSE), Mean Absolute Error (MAE), and Mean Absolute Percentage Error (MAPE) which are 0.990, 2.000, 0.323, and 13.848, respectively, prove this claim. Besides, in order to avoid the difficulties of working in the three-parameter space of RGB, converting to grayscale space has been applied which can estimate cell concentration with *R*^2^ = 0.99. Ultimately, an exponential correlation between RGB values and cell concentrations in lower amounts of biomass has been proposed based on Beer-Lambert law which can estimate activated sludge biomass concentration with *R*^2^ = 0.97 based on B index.

## Introduction

Use of activated sludge (AS) is the most common biological method for wastewater treatment (Gernaey et al., 2004). AS is a complex of viable microorganisms which is generally composed of mainly heterotrophic bacteria which can utilize organic matters, measured as biological oxygen demand (BOD) or chemical oxygen demand (COD), in the wastewater to survive and remove them from wastewater, consequently (Garakani et al., 2011; Ratkovich et al., 2013; Ju and Zhang, 2015). Quantitative and qualitative monitoring of different features of AS sometimes plays a key role in good operation of a wastewater treatment plant. Among all, controlling AS biomass concentration in the optimum range is most vital, because any deviation from the optimum range may result either in poor BOD removal or release of suspended solids into the effluent stream of the wastewater treatment plant which may cause microbial pollutions (Gernaey et al., 2001). Therefore, numerous attempts have been carried out for monitoring of biomass concentration in the AS systems described here.

Methods of biomass concentration measurement are categorized into two groups of direct and indirect techniques (Frasier et al., 2016). In direct techniques, the weight or cell numbers of the biomass will be measured directly, while in indirect techniques, a physical, chemical, or biological property which is depended on the biomass concentration will be used as a proxy for determination of biomass concentration (Ratkovich et al., 2013). Mixed liquor suspended solids (MLSS) is the most popular index which is used for biomass concentration in AS systems and is defined as the concentration of total suspended solids including biomass in a specific volume of sample taken from bioreactors (Martín-Pascual et al., 2015). Incapability of online measurement, noticeable errors of sampling, procedure of the measurement and having time-lag in reporting the results are the main drawbacks of direct methods such as MLSS measurement. Therefore, the attentions of the researchers of this field have been focused on development of appropriate indirect methods during last few years. Laser reflectance measurement (Expósito et al., 2017), measurement of ultrasound attenuation and backscattering (Rodriguez-Molares et al., 2014; Elvira et al., 2016), determination of cellular compounds and metabolites content such as adenosine triphosphate (ATP) (Abushaban et al., 2019), respirometry and measurement of oxygen uptake rate (OUR) in aerobic sludge (Garcia-Ochoa et al., 2010), flow cytometry (Brown et al., 2019), density measurement (Cano et al., 2014), and measurement of biomass electrical properties such as permittivity, capacitance, and impedance (Sarrafzadeh et al., 2005; Bobowski and Johnson, 2012; Zhang et al., 2012; Shariati et al., 2013; Pajoum-Shariati et al., 2014) are the most applied techniques which have been developed for biomass concentration measurement in different biological systems, especially bacterial ones such as activated sludge. A part of these methods successfully find their commercial place in several biotechnology processes but not in the biological wastewater treatment plants (Sarrafzadeh et al., 2005, 2015b). Because they are often considered as high-tech and complicated methods that need trained operators in addition to high capital consuming to be applied in wastewater plant.

Image processing has largely been used in recent years for quantitative analysis of biological systems such as yeast, bacteria and microalgae (Selinummi et al., 2005; Acevedo et al., 2009; Sarrafzadeh et al., 2015a). This method is generally based on analyzing the visual characteristics of the biological cultures such as color, light intensity, etc. through their images (Murphy et al., 2014). RGB analysis is one of the simplest and most common methods of image processing in which the intensities of three colors of red (R), green (G), and blue (B) of the image of culture will be extracted (Uyar, 2013). Different methods of image processing for monitoring of AS systems has found their positions among other methods of biomass quantification during recent years and analysis of sludge microscopic images has been used to estimate total suspended solids (TSS), sludge volume index (SVI), settling ability, sludge abnormalities, and disturbances (Amaral and Ferreira, 2005; Mesquita et al., 2009, 2011a, b, 2013; Amaral et al., 2013). However, using image processing for monitoring of AS cultures has been limited to the microscopic images of the cells and not macroscopic digital images. Microscope imaging not only makes online monitoring infeasible, but also the errors related to the operator and limited view scope of the microscope are normally significant. Besides, needing complex devices of microscopy will increase the costs of analysis in this technique. Therefore, using macroscopic digital imaging taken by simple camera or smart cell phone, not only facilitates the procedure of analysis, but also is a very good tool for online quantification of the biomass (Sarrafzadeh et al., 2015b).

Most of current methods of activated sludge monitoring needs sampling which increases inaccuracy due to human interference. On the other hand, image processing, is a non-destructive method with the potential of online application which can measure the activated sludge concentration accurately with minimum time lag. Moreover, the cheap methods of activated sludge monitoring (including drying and weighting, cytometry, etc.) are incapable of distinguishing between living and dead cells, however, combination of image processing with staining techniques or microscopic images can give valuable information about qualitative conditions of the cells in activated sludge culture (Saladra and Kopernik, 2016). Methods like permittivity or OUR measurement which are capable of provide a qualitative study of the activated sludge cells are either high-tech and need expensive devices and trained operators or are not generalizable and have limited area of application like OUR measurement which is only applicable in aerobic sludge.

In this study, it is tried to develop image processing as a non-invasive method for quantification of the activated sludge using macroscopic digital images and the advantages and limitations of this technique are discussed completely. The main purpose of this research is to study the feasibility of RGB analysis in order to measure the biomass concentration in AS systems.

## Materials and Methods

### Activated Sludge Samples and MLSS Measurement

AS was pre-cultivated in a 500 mL bottle with glucose as the carbon source for biomass growth. The inoculum size for precultivation was 172,000 cells per mL which was measured by plastic Neubauer (Improved DHC-N01, C-Chip, NanoEnTek, Korea) hemocytometer. The culture was continuously aerated with flowrate of 100 mL.min^–1^ to provide adequate mixing. After increasing the MLSS and reaching a desirable amount, the AS was injected to a 2 L Plexiglas fed-batch bioreactor with glucose as the carbon source and feeding rate of 3 g glucose (COD = 3200 mg.L^–1^) per 12 h (Rezaee et al., 2015; Leong et al., 2018). Then, sampling was carried out from the bioreactor at different times. A 100 mL plastic syringe was utilized for sampling AS from the reactor. In order to have a homogenous sample, representing the whole biomass in the bioreactor, sampling was carried out from three different heights of the bioreactor (surface, center, and bottom) with equal size and mixed together.

MLSS is the main index for AS biomass concentration which shows the weight of suspended particles of sludge in a specific volume. In order to measure the MLSS, a specific volume of the AS mixture is filtered in order to separate the liquid and solid phases. For filtration, pre-weighted filter papers (chm, 125 mm, Spain), Büchner funnel and vacuum pump (Value, single stage, VE 115N, China) are used. After filtration, the residue and filter are dried in an oven at 103–105°C for more than 1 h. Then, the dried filter and sludge are weighted and after subtraction from the weight of the filter paper, the dry weight of AS in the initial specific volume are determined. Calculation of the dry weight in 1 L of the mixture results in activated sludge MLSS in g.L^–1^ (Baird et al., 2017).

### Photography Conditions and Taking Images

**FIGURE 1:**
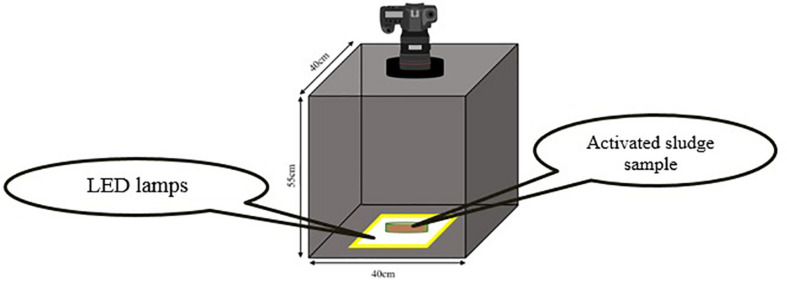
Schematic illustration of photography setup for taking images.

It is essential that all images be taken in the constant conditions. In order to preserve the consistency of photography conditions, all environmental parameters such as light intensity, distance between light camera and the samples and light source location which can interfere with the quality of images must remain the same. Therefore, a box with no entrance of light from the outside and with specific dimensions for obtaining best images was designed (Figure 1). In order to provide the necessary light for photography, cool white LED lamps were utilized. The box height is designed in a way that the best distance for the camera to be able to focus, be provided.

The camera used in this research was (Nikon D5300, Japan) equipped with (Nikon, 18–140 mm f/3.5-5.6 VR, Japan) lens. Since using camera zoom significantly affects the focal length of the lens and the image resolution consequently, all the images are acquired with the constant zoom of 140/18. The focal length of the lens under this circumstance will be 140 mm.

The samples must be poured into appropriate dishes with minimum light absorption and refraction coefficients. Besides, the height of dishes must be negligible in comparison to the distance between camera and the sample in order to avoid the influences of liquid height on the quality of images. For this purpose, glass petri dishes (diameter = 54 mm, PIREX, United Kingdom) were used in which the light refraction is negligible due to high transparency and very low thickness. Five milliliter of the sample is poured into the petri dishes in each test using sampler. Five milliliter was the minimum amount that could completely cover the surface of petri-dish with negligible height. Minimizing the height of the sample is the key point, since increasing the height, increases the light absorption logarithmically during diffusion through the sample and makes images darker than their real value and decreases measurement accuracy (Durduran et al., 2010). It is also important that the sample which is being poured into the dished be well mixed to be considered as a homogenous sample of the whole culture.

Diaphragm diameter, shutter speed and ISO number are three main parameters which can noticeably influence the resolution, light and colors intensities in the images (Radulescu and Vladareanu, 2017).

In order to keep the photography conditions constant during the tests, parameters of photography are set as Table 1. The criteria for choosing the photography conditions are totally qualitative and are based on the resolution and color intensities of the images. The logic of choosing these conditions depends on the range of MLSS changing and they will be chosen in a way that image resolution can cover the highest range of MLSS changes. It is more critical in very low and very high MLSS in which images are too bright and too dark, respectively. Therefore, the best solution is to make an estimation of the highest MLSS that may achieve during the process (about 15 g.L^–1^ in this research) and set the imaging conditions in this MLSS in a way that RGB analysis is possible and the image is not completely black. Then, the settings will be adjusted for MLSS ≤ 1 g.L^–1^ as well in order to avoid capturing too bright images which make RGB analysis infeasible and the image is completely white. Since, the criterion for evaluating the quality of images is qualitative (not quantitative) statistical analysis and design of experiments is infeasible for reducing the number of images and this innovative method based on evaluation in boundaries must be followed. However, defining a quantitative criterion for quality of images in the process of RGB analysis can be a suggestion for further studies.

**TABLE 1:** Photography conditions and camera settings for taking images.

Parameter	Value
f number	5.6
Shutter time (s)	1/160
ISO	100
Zoom	140/18
Focal length (mm)	140

In this research, the optimum conditions for acquiring the most appropriate images are obtained (Table 1) with 9 different images. In activated sludge applications, since 15 g.L^–1^ is usually the darkest concentration that can be reached, these settings can be applied to the most of other cases in which image processing is being used for MLSS measurement in activated sludge systems.

For each sample, imaging are conducted three times and the final value of RGB is considered as the average value of the RGB of all three images.

### Image Processing Procedure

RGB analysis of AS means extraction of red, green and blue intensities in the images of sludge and correlate them to a quantitative parameter of the studied system such as MLSS. RGB extraction will be carried out using ImageJ^®^ which is an open-source software for image management, editing and processing (Schneider et al., 2012). In RGB model, a three-vector coordination for each pixel of the image will be defined based on R, G and B which shows the color intensity of the pixel. Each color is defined as an 8-bit data package and considering three vectors of R, G and B for each color, 8^3^ = 256 different numbers for all of the colors existing in an image are designated. Therefore, RGB numbers are in the range between 0 and 255 where (0,0,0) and (255,255,255) are black and white respectively (Kelda and Kaur, 2014). However, dealing with three-parameter space of RGB is not always simple and it is usually more desirable to work in a single-parameter space. Grayscale is a conversion which is commonly used for changing RGB space to a single-parameter space (Bala and Braun, 2003).

There are various equations and procedures for converting RGB space to grayscale which has been used based on the application in which image processing is used. Córdoba-Matson et al. (2010) have proposed a new approach for grayscale conversion in order to determine the cell numbers in microalgae cultures by image processing. In this approach, Eqs 1–4 are used to convert the color image to grayscale:

(1) $$\mathrm{Grayscale}=K_{R}R+K_{G}G+K_{B}B$$

K_R_, K_G_, and K_B_ are gray coefficients for red, green and blue respectively which are calculated as following:

(2) $$K_{R}=M_{R}/\left(M_{R}+M_{G}+M_{B}\right)$$

(3) $$K_{G}=M_{G}/\left(M_{R}+M_{G}+M_{B}\right)$$

(4) $$K_{B}=M_{B}/\left(M_{R}+M_{G}+M_{B}\right)$$

where M_R_, M_G_, and M_B_ are the slopes of linear fitting of R, G, and B values vs. desired parameter (MLSS in this case).

## Results and Discussion

**FIGURE 2:**
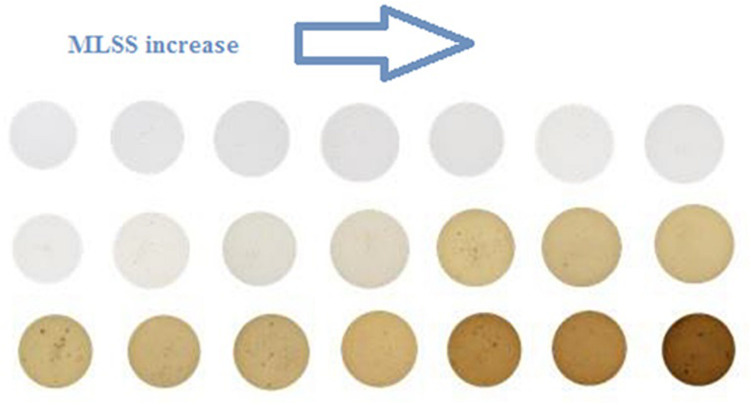
Color changing with MLSS increase in activated sludge.

**FIGURE 3:**
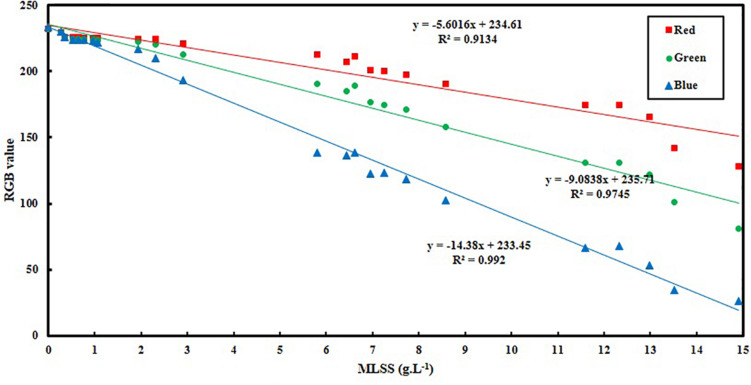
Variation of RGB values vs. activated sludge MLSS.

Figure 2 shows the color variation with increase in MLSS in activated sludge. It helps the reader to have a better understanding about the results of RGB analysis and trends in Figure 3. In other words, Figure 2 is the realization of Figure 3 for generating a visual image about what is going on in reality with changing RGB. Moreover, it is obvious from Figure 2 that the color of the culture noticeably changes with MLSS and it proves that RGB analysis can be an appropriate technique for studying activated sludge MLSS variation. It can be seen in Figure 2 that the samples get darker with increase in MLSS. Therefore, it is expected that with MLSS growth, RGB values decrease consequently.

Figure 3 and Table 2 show the variation of RGB values with MLSS in AS. RGB acquisition has been carried out in 21 different samples with different MLSS. Each sample was photographed three times and RGB data of each sample is the average of the RGB data of these three images. Totally, 63 images were taken and processed whose results are provided in Figure 3.

**TABLE 2:** MLSS and RGB data in this study.

MLSS (g.L^–^^1^)	R	G	B
0	232.08	232.09	233.6
0.263	229.51	229.35	229.71
0.34	225.81	225.51	225.66
0.527	225.85	224.62	223.85
0.644	225.58	224.5	223.7
0.772	225.4	224.41	223.49
0.966	225.03	224.11	223.001
1.053	224.97	223.36	221.49
1.931	224.46	222.18	216.78
2.318	224.26	220.21	210.13
2.897	221.07	212.37	193.21
5.795	212.44	190.33	138.33
6.623	211.21	188.84	138.42
6.439	207.118	184.67	136.23
6.954	200.72	176.9	122.58
7.243	200.35	174.85	123.05
7.726	197.4	170.91	118.35
8.585	190.2	157.97	102.74
11.59	174.36	130.76	66.12
12.33	174.56	131.07	67.7
12.98	165.33	121.65	53.14
13.52	142.19	101.2	34.193
14.901	128.15	81.26	26.2

It can be concluded from Figure 3 that B is the best vector for MLSS estimation in AS systems which shows *R*^2^ = 0.99. Since, blue color is located at the end of electromagnetic spectra and very close to UV region, its absorption coefficient is higher in comparison to green and red and its variation is more intensive consequently. Therefore, when the range of variation of MLSS in AS mixture gets wider (0–15 g.L^–1^), B will be fitted more accurately for studying the system. This range of MLSS is the most applicable is AS systems. Conventional activated sludge systems cannot operate efficiently in concentrations higher than 5 g.L^–1^ and their operational MLSS lies between 1 and 5 g.L^–1^ with optimal value of 3–4 g.L^–1^. On the other hand, membrane bioreactors (MBRs) are usually operated at higher concentrations (5–15 g.L^–1^) (Sari Erkan et al., 2018). Therefore, the method is accurate enough for being applied in both conventional and MBR systems of AS.

As it is mentioned before, in order to avoid dealing with three-parameter space of RGB, grayscale conversion is usually used. According to the results of Figure 3, Table 3 is obtained which shows the grayscale conversion coefficients for activated sludge MLSS based on Eqs 1–4. Linear fitting has been applied using RGB data and MLSS and the slopes are reported as M_R_, M_G_ and M_B_. Then K_R_, K_G_ and K_B_ are defined using M data and according to Eqs 2–4. Replacing K data in Eq. 1 results in Eq. 5.

**TABLE 3:** Grayscale conversion coefficients for activated sludge MLSS.

Coefficient	Value
M_R_	–5.3117
M_G_	–8.9099
M_B_	–14.404
K_R_	0.185
K_G_	0.311
K_B_	0.503

**FIGURE 4:**
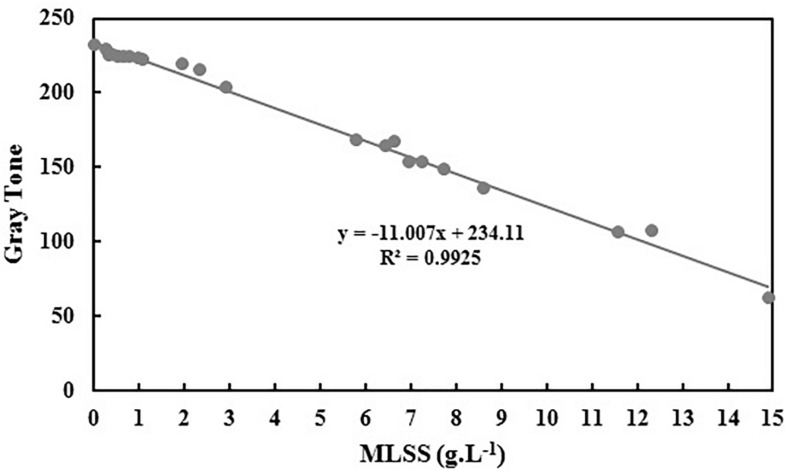
Variation of gray tone vs. activated sludge MLSS.

Using the data provided in Table 3, equation 1 is rewritten as equations 5 and the graph of gray tone variation with MLSS is obtained as Figure 4.

(5) Grayscale=0.185R+0.311G+0.503B

Equation 5 is the grayscale conversion equation for AS which converts the RGB image into the grayscale image.

The slope and intercept of the achieved correlation for MLSS estimation using gray tone is so similar to B and the R^2^ coefficients are almost the same. Therefore, it can be concluded that choosing B as the best vector for MLSS estimation in AS systems is rational and accurate.

**FIGURE 5:**
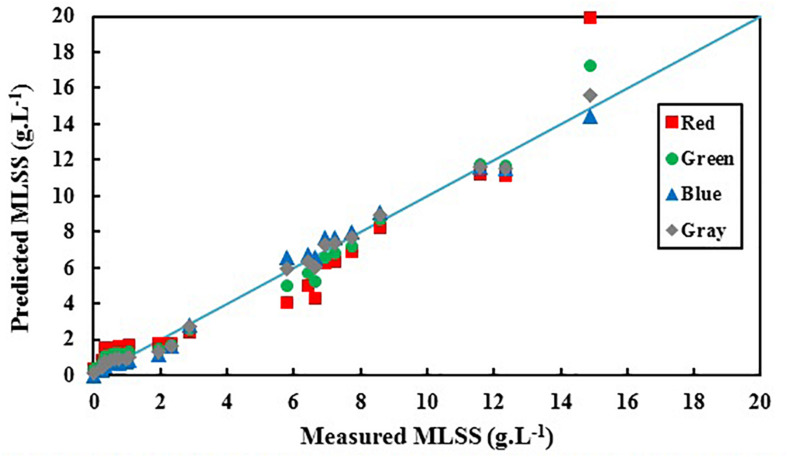
Predicted activated sludge MLSS based on red, green, blue, and gray colors vs. actual biomass MLSS.

Figure 5 shows the deviation between predicted values of activated sludge MLSS by RGB analysis and the actual measured values.

It is obvious in Figure 5 that predicted MLSS based on B shows the least deviation from the actual results comparing R, G and gray. Table 4 shows the summary of the results of statistical analysis for R, G, and B in order to choose the most accurate index for activated sludge MLSS estimation.

**TABLE 4:** Statistical deviations of MLSS estimation based on RGB indices individually.

Index	RMSE*	MAE**	MAPE*** (%)	*R*^2^
R	6.714	1.058	67.136	0.900
G	3.547	0.605	42.584	0.970
B	2.000	0.323	13.848	0.990
Gray	0.045	0.291	20.649	0.992

*Root mean square error. **Mean absolute error. ***Mean absolute percentage error.

According to the data of Table 3, blue index (B) shows the least errors and highest R^2^ and least errors which makes it the most accurate index for activated sludge MLSS estimation which has been proposed earlier. Also, gray shows sufficient accuracy in activated sludge MLSS measurement which may come helpful considering its cumulative nature.

**FIGURE 6:**
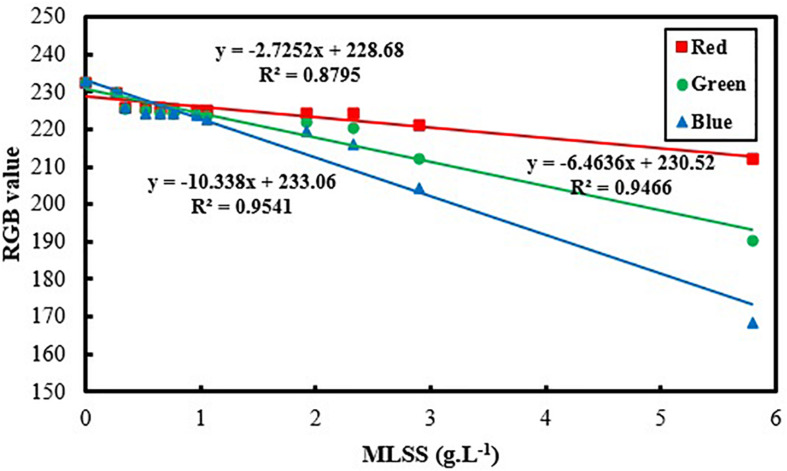
Variation of RGB values vs. activated sludge MLSS in low ranges (MLSS < 6 g.L^– 1^).

In the lower ranges of MLSS which are common in conventional activated sludge systems (< 6 g.L^–1^), the changes in darkness of images are not so intensive, so that G also shows the proficient accuracy in MLSS measurement, while the deviation in R accuracy increases, because G is in the middle zone of the light spectrum and is less sensitive to harsh changes. Still, B shows the higher R^2^ value and confirms the claim that B is most accurate vector in all ranges of MLSS, since it shows the least deviation from the actual data (Figure 5) in all ranges. Figure 6 shows variation of RGB data with MLSS in low ranges.

As it is mentioned, the image which is recorded by the camera is the result of the light which has been diffused through the AS sample and reached to the camera. In other words, the intensity of the light which is reached to the camera controls the image qualities including colors intensity. Therefore, it is predicted that within the range of biomass concentration where light absorption is negligible in comparison to light diffusion, variation of RGB values vs. MLSS be similar to variation of light intensity.

**FIGURE 7:**
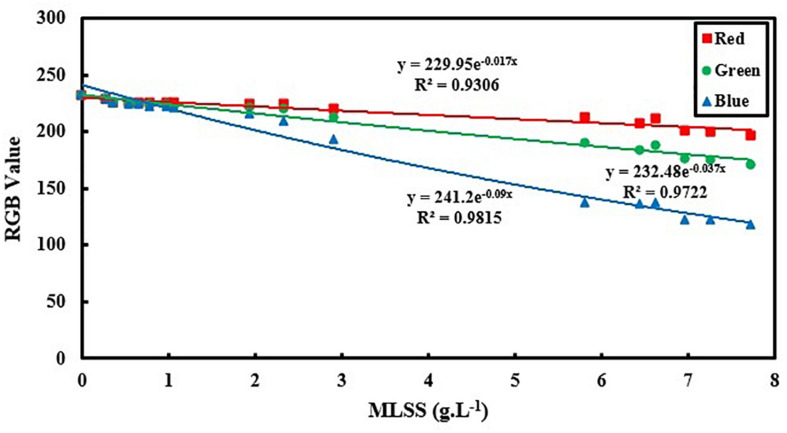
Exponential relation between RGB values and activated sludge MLSS based on optical diffuse theory.

According to the theory of optical diffuse, light intensity changes exponentially with the differences in cell concentration (Ripoll et al., 2003). Hence, RGB must change exponentially with MLSS in lower amounts of cell concentration, since with increase in MLSS the culture gets more turbid and light absorption increases consequently. As a result to light absorption increase, the light scattering in the AS culture increases and deviation from the theory of optical diffuse increases subsequently. In this section, it is try to test the results of this study to see if they are in accordance with the assumptions of optical diffuse. Figure 7 shows the exponential trend of RGB values with MLSS.

As it is shown in Figure 7, for the MLSS < 8 g.L^–1^, exponential relation can accurately analyze the AS system using all three vectors of R, G and B. However, B still shows the greatest accuracy in comparison to R and G. In lower MLSS ranges (MLSS < 8 g.L^–1^), the diffuse approximation is valid for light transfer and the assumption of a logarithmic correlation between RGB data and MLSS is physically logical which is in accordance with optical diffuse assumption (Gibson and Dehghani, 2009). It also proves proficiency of B as the most appropriate vector in all ranges of MLSS. However, comparing the results of Figure 6 with Figure 3 reveals that the linear fitting developed in this manuscript for MLSS measurement using B is still more accurate that logarithmic fitting which is probably due to some simplifying assumptions in optical diffuse approximation which may not be applicable in activated sludge environment and reduce the accuracy of measurement.

## Conclusion

Biomass concentration is the key parameter in activated sludge wastewater treatment systems which must be monitored during the process in order to reassure the optimum performance of treatment system. In this research, a new method is proposed for measurement of activated sludge concentration based on macroscopic imaging and RGB analysis. The method proposed in this study shows acceptable results in activated sludge quantification without needing expensive or complicated devices or skills. However, it is early stages of applying this method for online monitoring of activated sludge cultures and some considerations must be taken into account in order to not only make the method capable of online measurement but also to provide the situation of qualitative study of the system which will be the main focus of our future research. Applying this method in large industrial scales (e.g., a wastewater treatment plant) needs different pre-requisites most of which are addressed in this research such as criteria for image acquisition, choosing the most accurate vector for image analysis, fitting equation, and the statistical analysis of the method. Therefore, the results of this research are essential for applying this method. Consequently, considering the results of current study, measurement of other concentration-related parameters of activated sludge such as its settling properties by image processing and RGB analysis can be good subjects for further studies of the researchers of this field. In comparison to other methods of MLSS quantification in activated sludge systems, image processing has showed to be cheaper since there is no need to high-tech devices in this method. It has a great potential for applying online which makes it a very appropriate candidate for commercializing evaluations. Since, the process of sampling and sample preparations is almost eliminated in this method, human errors are minimized which brings acceptable accuracy for this method. Moreover, it has the capacity of combination with microscopic imaging which provides the capability of simultaneous qualitative and quantitative study of activated sludge systems and can be a subject to further studies in this field.

## Data Availability Statement

The authors confirm that all data supporting the findings of this study are available within the article.

## Author Contributions

HA: experiments, data analysis, data validation, software application, and writing – original draft. M-HS: conceptualization, supervision, project administration, and writing – reviewing and editing. Both authors contributed to the article and approved the submitted version.

## Conflict of Interest

The authors declare that the research was conducted in the absence of any commercial or financial relationships that could be construed as a potential conflict of interest.

## Nomenclature

**Table:**

Terminology	Abbreviation	Terminology	Abbreviation
Activated Sludge	AS	Green	G
Biological Oxygen Demand	BOD	Blue	B
Chemical Oxygen Demand	COD	Total Suspended Solids	TSS
Mixed Liquor Suspended Solids	MLSS	Sludge Volume Index	SVI
Oxygen Uptake Rate	OUR	Ultra Violet	UV
Red	R	Membrane Bioreactor	MBR
